# Neutrophil gelatinase-associated lipocalin as a prognostic biomarker of severe acute respiratory distress syndrome

**DOI:** 10.1038/s41598-022-12117-4

**Published:** 2022-05-12

**Authors:** Eunjeong Son, Woo Hyun Cho, Jin Ho Jang, Taehwa Kim, Doosoo Jeon, Yun Seong Kim, Hye Ju Yeo

**Affiliations:** 1 Division of Pulmonary, Allergy, and Critical Care Medicine, Department of Internal Medicine, Pusan National University Yangsan Hospital, Yangsan, Republic of Korea; 2 Department of Pulmonology and Critical Care Medicine, Research Institute for Convergence of Biomedical Science and Technology, Pusan National University Yangsan Hospital, Geumo-ro 20, Beomeo-ri, Mulgeum-eup, Yangsan-si, Gyeongsangnam-do 626-770 Republic of Korea

**Keywords:** Biomarkers, Medical research

## Abstract

Neutrophil gelatinase-associated lipocalin (NGAL) is produced in the bronchial and alveolar cells of inflamed lungs and is regarded as a potential prognostic biomarker in various respiratory diseases. However, there are no studies on patients with acute respiratory distress syndrome (ARDS). NGAL levels in serum and bronchoalveolar lavage (BAL) were measured at baseline and on day 7 in 110 patients with ARDS. Baseline NGAL levels were significantly higher in ARDS patients than in healthy controls (serum 25 [14.5–41] vs. 214 [114.5–250.3] ng/mL; BAL 90 [65–115] vs. 211 [124–244] ng/mL). In ARDS, baseline NGAL levels in serum and BAL were significantly higher in non-survivors than in survivors (p < 0.001 and p = 0.021, respectively). Baseline NGAL levels showed a fair predictive power for intensive care unit (ICU) mortality (serum area under the curve (AUC) 0.747, p < 0.001; BAL AUC 0.768, p < 0.001). In a multivariate Cox regression analysis, the baseline serum NGAL level (> 240 ng/mL) was significantly associated with ICU mortality (hazard ratio [HR] 5.39, 95% confidence interval [CI] 2.67–10.85, p < 0.001). In particular, day 7 NGAL was significantly correlated with day 7 driving pressure (serum r = 0.388, BAL r = 0.702), and 28 ventilator-free days (serum r = − 0.298, BAL r = − 0.297). Baseline NGAL has good prognostic value for ICU mortality in patients with ARDS. NGAL can be a biomarker for ventilator requirement, as it may be indicative of potential alveolar epithelial injury.

## Introduction

Acute respiratory distress syndrome (ARDS) is the most severe form of acute lung injury, with a mortality rate between 34 and 60%^1,2^. The severity of ARDS is often assessed using the PaO_2_/FiO_2_ ratio, but the prognostic value of this variable is limited^3^. Recently, several biomarkers have been suggested for predicting the prognosis and therapeutic response of ARDS^4^. However, none of these have been used in clinical practice. Other clinical predictors such as the sequential organ failure assessment (SOFA) and Acute Physiology and Chronic Health Evaluation (APACHE) score have been used, but they are complex and often not followed^5^. Simplified and more accurate predictors are still lacking, and many studies on novel biomarkers are ongoing^4,6,7^.

Neutrophil gelatinase-associated lipocalin (NGAL) is a 25-kDa protein released from neutrophils and epithelial cells and may be used as a marker for various inflammatory diseases^8^. Conventionally, NGAL has been used as a marker of acute kidney injury (AKI), as it rapidly increases in renal tubular cells^9^. NGAL is also found in bronchial goblet cells and alveolar type II pneumocytes, with increased levels in these cells in inflamed lungs^10^. In a previous study, we found that NGAL is a potential biomarker of ventilator-induced lung injury (VILI) in a rat model^11^. Another study suggested that NGAL may be produced during alveolar cell damage triggered by barotrauma^12^. Recently, NGAL has been suggested as a valuable biological marker for predicting the severity and prognosis of patients with various respiratory diseases^13–15^.

Despite evidence that NGAL is a potential biomarker for alveolar or bronchial epithelial cell damage, the concentration of NGAL required for predicting the outcome of ARDS and the severity of lung injury is still unknown. In this study, we evaluated NGAL levels from serum and bronchial alveolar lavage (BAL) as a potential biomarker for predicting mortality in patients with severe ARDS.

## Methods

### Study design and patients

**Figure 1:**
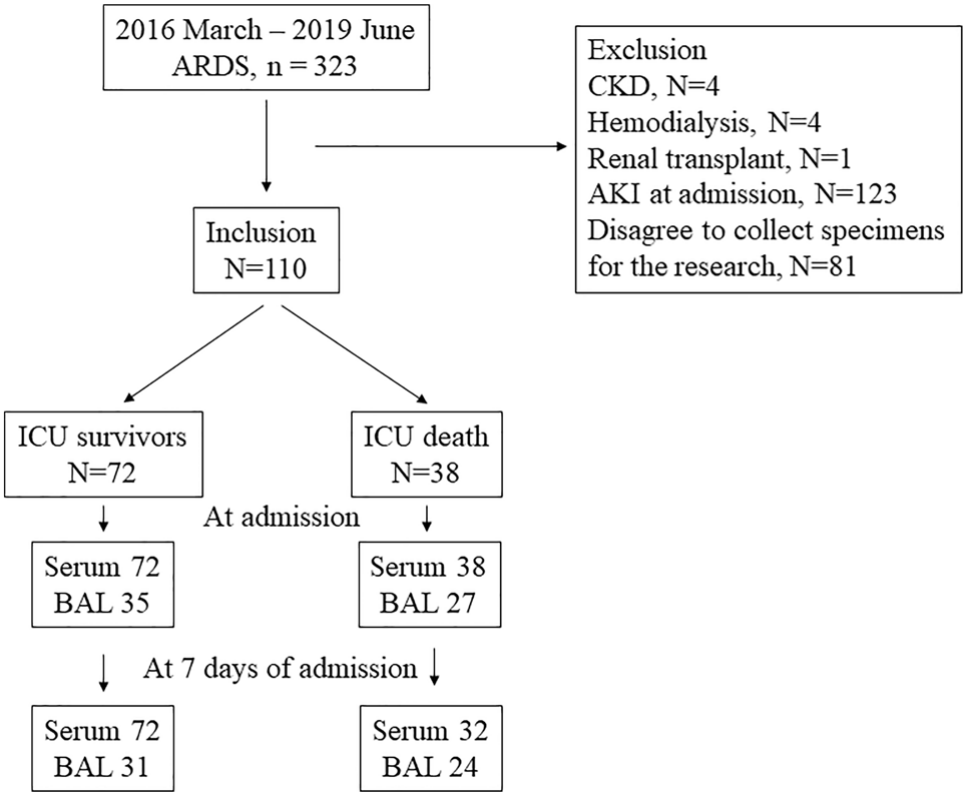
Flowchart of patient inclusion. During the study period, 323 patients were treated for acute respiratory distress syndrome, of which 110 were included in this study. *ARDS* acute respiratory distress syndrome, *CKD* chronic kidney disease, *AKI* acute kidney injury, *ICU* intensive care unit, *BAL* bronchial alveolar lavage.

Patients aged > 18 years, who were treated for ARDS in the intensive care unit (ICU) from March 2016 to June 2019, were assessed for eligibility in this study. ARDS was defined based on the Berlin definition^3^. Patients with known chronic kidney disease, patients on hemodialysis, renal transplant recipients, and patients with AKI at admission according to Kidney Disease: Improving Global Outcomes guidelines were excluded^16^ (Fig. 1). Finally, 110 patients were included. Serial specimens, including serum and BAL, were obtained from the Pusan National University Yangsan Hospital (PNUYH) biobank; the first within 6 h of admission to the ICU, and the second 7 days after admission. The biobank provided well-banked samples and clinical data. Patients were divided into two groups: survivors at discharge from the ICU, and non-survivors. We retrospectively analyzed the correlations between ICU mortality and the NGAL levels in serum and BAL. This single-center, retrospective cohort study was approved by the Institutional Review Board (IRB) of Pusan National University Yangsan Hospital (PNUYH) (05-2019-185). Informed consent was waived by the PNUYH IRB. The study was performed in accordance with all relevant guidelines and regulations and conducted in accordance with the Declaration of Helsinki.

### Data collection

Demographics, renal replacement therapy (RRT), main diagnosis, and laboratory data were collected retrospectively. SOFA, APACHE II, lung injury, and Charlson comorbidity scores, arterial blood gas analysis, C-reactive protein (CRP), creatinine, estimated glomerular filtration rate, duration of extracorporeal membrane oxygenation (ECMO), and ventilator requirement were included. None of the patients had previously received treatment for ARDS. Ventilator-free days (VFDs) were defined as the number of days from successful weaning to day 28; patients who died before weaning were deemed to have no VFDs.

### Measurement of NGAL, interleukin (IL)-1b, and tumor necrosis factor (TNF)-α levels in serum and BAL

Blood samples were collected from 110 patients at baseline and 104 patients at day 7. Blood samples were collected in a serum separate test (SST) tube (Greiner Bio-One, Austria). To test the levels of NGAL and other cytokines, the serum samples were centrifuged at 2500*g* for 20 min, 1000*g* for 10 min, and 2000*g* for 10 min, within 30 min of collection. Aliquots of serum were stored at − 80 °C to facilitate batch analysis. Serum levels of NGAL, IL-1β, and TNF-α were determined in accordance with the manufacturer’s instructions using enzyme immunoassays, namely the Human NGAL ELISA Kit (BioPorto Diagnostics A/S, Gentofte, Denmark), the Human IL-1b Pre-Coated ELISA Kit (BioGems, Westlake Village, CA, USA), and the Human TNF-α Pre-Coated ELISA Kit BGK01375 (BioGems). BAL samples were collected from 62 patients at baseline and 55 patients on day 7. Aliquots of BAL were stored at − 80 °C to facilitate batch analysis. The levels of NGAL, IL-1β, and TNF-α in BAL were determined in accordance with the manufacturer’s instructions, as mentioned above.

### Statistical analyses

All statistical analyses were performed using SPSS Statistics Software for Windows, Version 26.0. (IBM Corp., Armonk, NY, USA), or MedCalc Statistical Software version 18 (MedCalc Software bvba, Ostend, Belgium). Numerical results are expressed as mean ± standard deviation (SD), or median (interquartile range), as appropriate. Continuous variables in the survivor and non-survivor groups were compared using the independent t-test (parametric values), or Mann–Whitney U test (non-parametric values), as appropriate. The chi-squared test or Fisher’s exact test was used to compare categorical variables. In all analyses, a p-value < 0.05 was considered statistically significant. A receiver operating characteristic (ROC) curve was used to assess the prognostic function of baseline NGAL levels for predicting ICU mortality. Cox proportional hazards regression analysis was used to determine ICU mortality. The ROC curve analysis was used to identify the optimal cut-off values of NGAL in BAL and serum according to the Youden index. ICU mortality was analyzed using a Kaplan–Meier curve according to baseline NGAL levels in serum and BAL. In Supplementary Fig. 1, BAL (A) and serum (B) NGAL levels were expressed as fold increases in NGAL levels (each value/mean of control).

### Ethics approval and consent to participate

This single-center, retrospective cohort study was approved by the Institutional Review Board of Pusan National University Yangsan Hospital (PNUYH) (05-2019-185). Informed consent was waived by the PNUYH IRB because of the retrospective nature of the study.

## Results

### Baseline characteristics

A total of 110 patients with ARDS were included in this study. Patients were divided into two groups: ICU survivors, 72 (65.5%) and non-survivors, 38 (34.5%). Age, sex, body mass index, lung injury score, and initial PaO_2_/FiO_2_ ratio did not show any significant differences between the two groups (Table 1). There were no significant differences in the causes of ARDS between the two groups. In addition, the use of vasopressors, the application of ECMO, and the levels of CRP and creatinine at the time of hospitalization were not significantly different between the two groups. The initial SOFA and APACHE II scores were significantly higher in the non-survivors than in the survivors (SOFA 9.9 vs. 12.5, p < 0.001; APACHE II 18.0 vs. 21.8, p = 0.011). In terms of respiratory support, there was no significant difference in median ventilator duration (13.5 vs 15 days, p = 0.578). In addition, there was no significant difference in the median ECMO duration between the two groups (7 vs. 8 days, p = 0.962). However, the median driving pressure on days 1 and 7 was significantly higher in non-survivors than in survivors (day 1: 24 vs. 26, p = 0.006; day 7: 22 vs. 24, p = 0.006). The requirement for RRT during treatment was significantly higher in non-survivors than in survivors (12.5% vs. 28.9%, p = 0.033).

**Table 1:** Baseline characteristics according to intensive care units mortality.

	Survivor (n = 72)	Death (n = 38)	*p*
Age, year	58.9 ± 17.0	65.0 ± 13.5	0.062
Elderly (≥ 65 years)	29 (40.3)	20 (52.6)	0.215
Male	38 (52.8)	26 (68.4)	0.114
BMI, kg/m^2^	23.0 ± 4.9	22.6 ± 3.7	0.684
SOFA	9.9 ± 2.9	12.5 ± 1.9	< 0.001
APACHE II	18.0 ± 6.5	21.8 ± 7.8	0.011
LIS	3.4 ± 0.3	3.5 ± 0.3	0.239
PF ratio	117.7 ± 58.0	107.2 ± 44.5	0.331
**Cause of ARDS**			0.535
Pneumonia	63 (87.5)	33 (86.8)	
Non-pulmonary sepsis	3 (4.2)	0	
ILD	5 (6.9)	4 (10.5)	
Other	1 (1.4)	1 (2.6)	
Initial vasopressor use	55 (76.4)	33 (86.8)	0.192
CRRT during treatment	9 (12.5)	11 (28.9)	0.033
ECMO use	44 (61.1)	19 (50.0)	0.263
CRP	17.8 ± 12.1	18.3 ± 12.1	0.842
Creatinine, mg/dL	1.1 ± 0.6	1.2 ± 0.6	0.493
MV duration^a^	13.5 (7–25.3)	15 (7–30.0)	0.578
ECMO duration^a^	7 (4–16.5)	8 (0.8–20.5)	0.962
ICU duration	24.9 ± 24.0	23.1 ± 18.8	0.690
**Driving pressure**			
Day 1^a^	24 (20–28)	26 (24–30)	0.006
Day 7^a^	22 (19–24)	24 (22–27.3)	0.006

*BMI* body mass index, *SOFA* Sequential Organ Failure Assessment, *APACHE II* Acute Physiology and Chronic Health Evaluation II, *LIS* lung injury scores, *PF ratio* PaO_2_/FiO_2_ ratio, *ARDS* adult respiratory distress syndrome, *ILD* interstitial lung disease, *ECMO* C-reactive protein, *MV* mechanical ventilator, *ICU* intensive care unit.

^a^Data are presented as median (interquartile range). Other data are presented as mean ± standard deviation or N (%).

### NGAL and inflammatory cytokines

**Figure 2:**
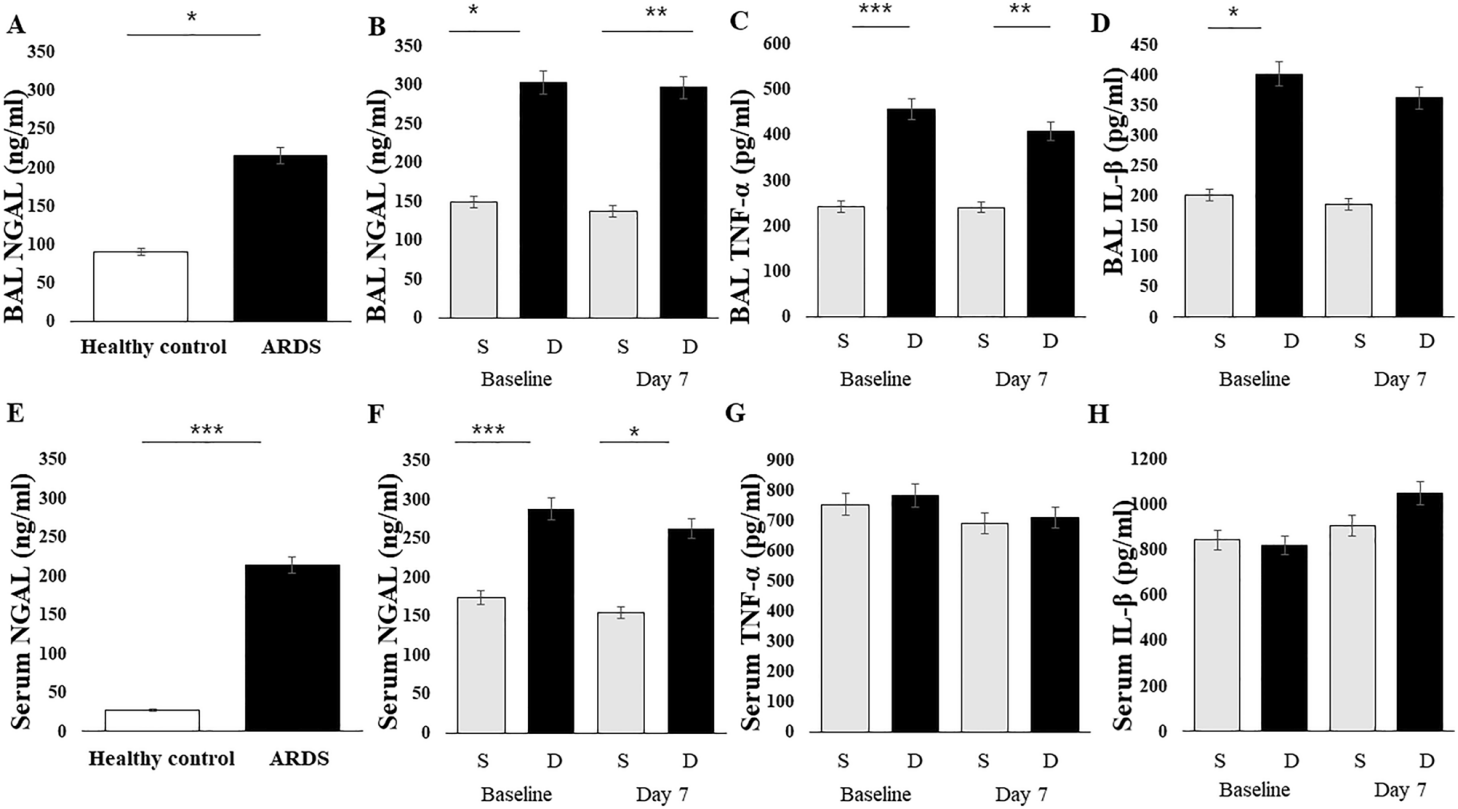
NGAL and inflammatory markers. Data presented as mean ± se. BAL specimens were collected at baseline from 62 patients (35 survivors and non-survivors 27) and on day 7 from 55 patients (31 survivors and non-survivors 24). *BAL* bronchoalveolar lavage, *NGAL* neutrophil gelatinase-associated lipocalin, *TNF* tumor necrosis factor, *IL* interleukin. *p < 0.05, **p < 0.01, ***p < 0.001.

Baseline NGAL levels were significantly higher in ARDS patients than in healthy controls (n = 5) (serum 25 vs. 214 ng/mL, p = 0.023; BAL 90 vs. 211 ng/mL, p = 0.026, Fig. 2A,E). In ARDS, baseline NGAL levels were significantly higher in non-survivors than in survivors (serum 184 vs. 247.5 ng/mL, p < 0.001; BAL 130 vs. 240 ng/mL, p = 0.021) (Fig. 2B,F). IL-1β and TNF-α (pg/mL) in BAL were significantly higher in non-survivors than in survivors (p < 0.001, p = 0.011; Fig. 2C,D). However, there were no significant differences in serum TNF-α and IL-1β levels between the two groups (Fig. 2G,H).

**Figure 3:**
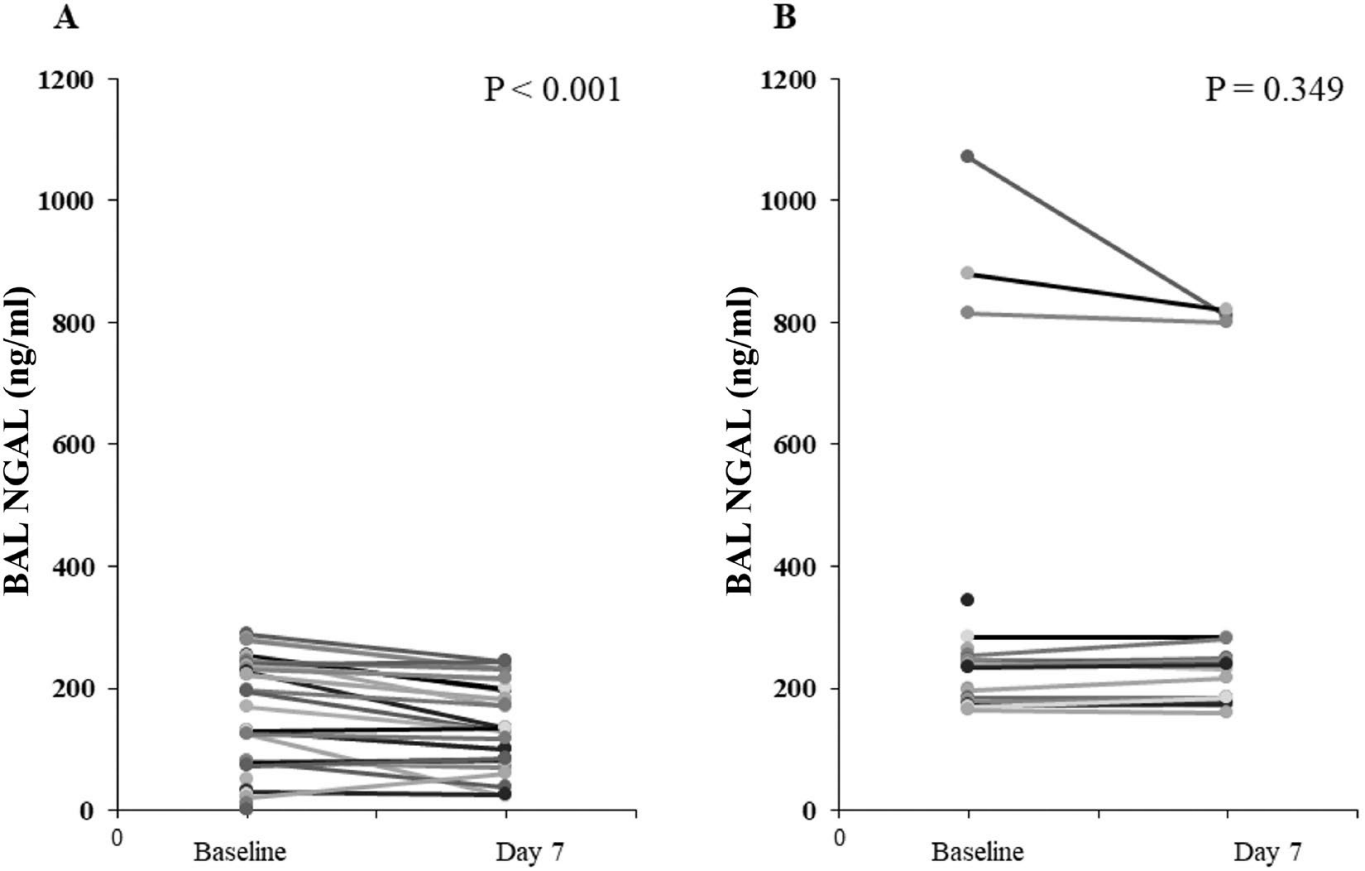
NGAL level of baseline and day 7 in bronchoalveolar lavage. A paired t-test was used to compare baseline BAL NGAL and day 7 BAL NGAL [(**A**) survivors (**B**) non-survivors]. *BAL* bronchoalveolar lavage, *NGAL* Neutrophil gelatinase-associated lipocalin.

The difference in BAL NGAL values from baseline to day 7 was higher in survivors compared to non-survivors. In the survivors, the BAL NGAL value decreased from baseline to day 7 as median − 26, and in non-survivors, the value increased from baseline to day 7 as median 0.5 (p = 0.002). Figure 3 shows the changes in BAL NGAL at baseline and day 7 in each group. Otherwise, there were no significant differences in serial changes in serum NGAL levels between the two groups.

### Correlation of NGAL level with severity of ARDS and parameters of mechanical ventilation

Baseline NGAL levels were significantly correlated with the initial SOFA score (BAL r = 0.274, p = 0.031, serum; r = 0.231, p = 0.015). In addition, these levels were inversely correlated with 28 VFDs (serum r = − 0.296, p = 0.002, BAL r = − 0.250, p = 0.050). In line with this, day 7 NGAL was significantly associated with specific parameters of mechanical ventilation, such as day 7 driving pressure (serum r = 0.388, p < 0.001, BAL r = 0.702, p < 0.001), and 28 VFDs (serum r = − 0.298, p = 0.002, BAL r = − 0.297, p = 0.028).

### NGAL and prediction of ICU mortality

**Figure 4:**
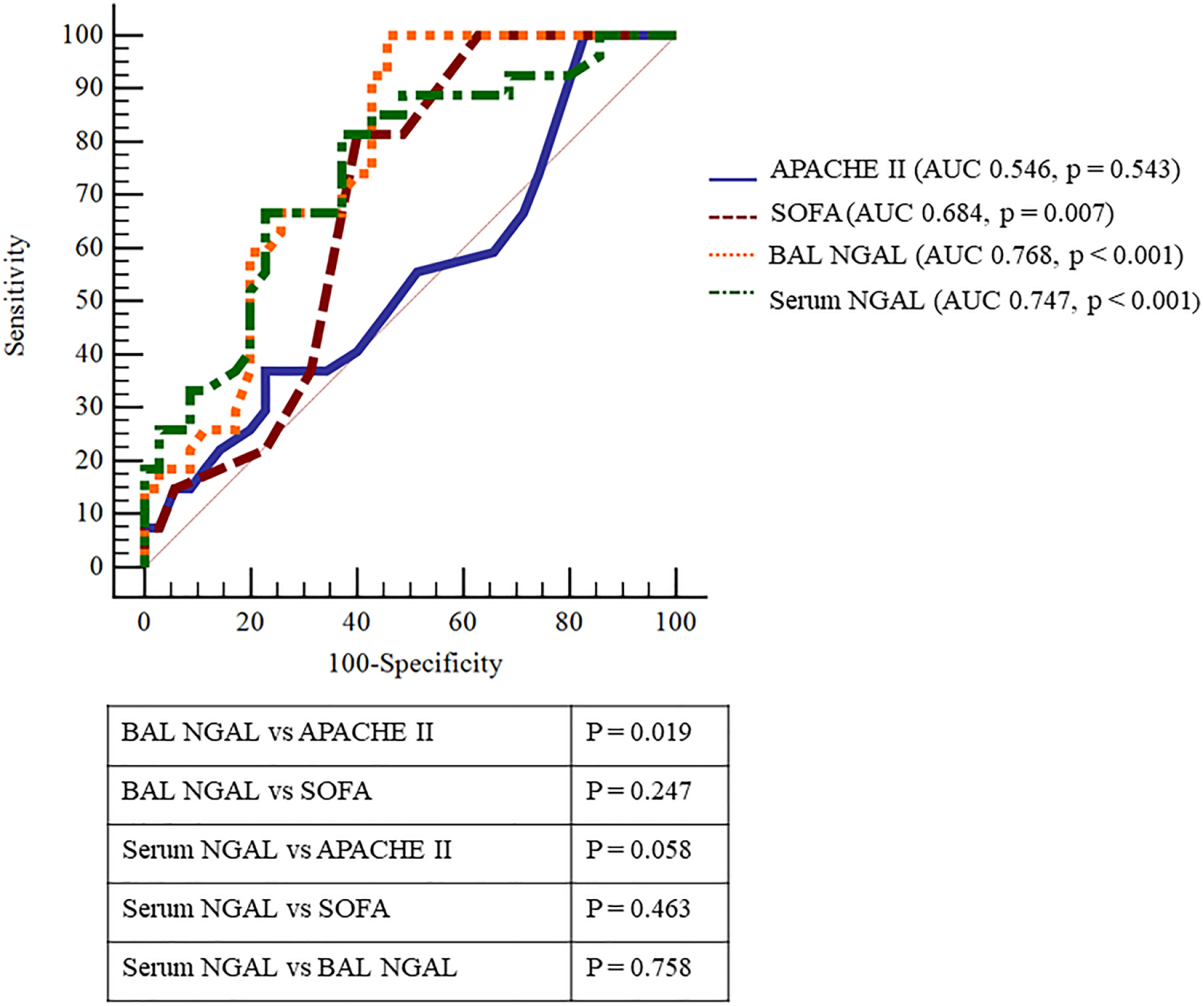
Receiver operating characteristic curve for intensive care unit mortality. At baseline, BAL NGAL (AUC 0.768, 95% CI 0.643–0.866, p < 0.001) and serum NGAL (AUC 0.747, 95% CI 0.621–0.849, p < 0.001) levels showed fair predictive power. SOFA score (AUC 0.684, 95% CI 0.554–0.796, p = 0.007) also showed significant predictive power. Baseline NGAL levels showed comparable predicted power compared to SOFA scores (serum P = 0.463, BAL p = 0.247). In addition, serum NGAL showed a predicted power comparable to that of BAL NGAL (p = 0.758). *AUC* area under the curve, *BAL* bronchoalveolar lavage, *NGAL* neutrophil gelatinase-associated lipocalin, *APACHE* *II* Acute Physiology and Chronic Health Evaluation II, *SOFA* sequential organ failure assessment.

ROC curves of the association of NGAL and clinical markers with ICU mortality are shown in Fig. 4. At baseline, BAL NGAL (area under the curve (AUC) 0.768, 95% CI 0.643–0.866, p < 0.001), and serum NGAL (AUC 0.747, 95% CI 0.621–0.849, p < 0.001) levels showed fair predictive power, comparable to the SOFA scores (AUC 0.684, 95% CI 0.554–0.796, p = 0.007). However, APACHE II scores did not significantly predict ICU mortality (AUC 0.546, 95% CI 0.414–0.673, p = 0.543).

### Factors associated with ICU mortality

**Figure 5:**
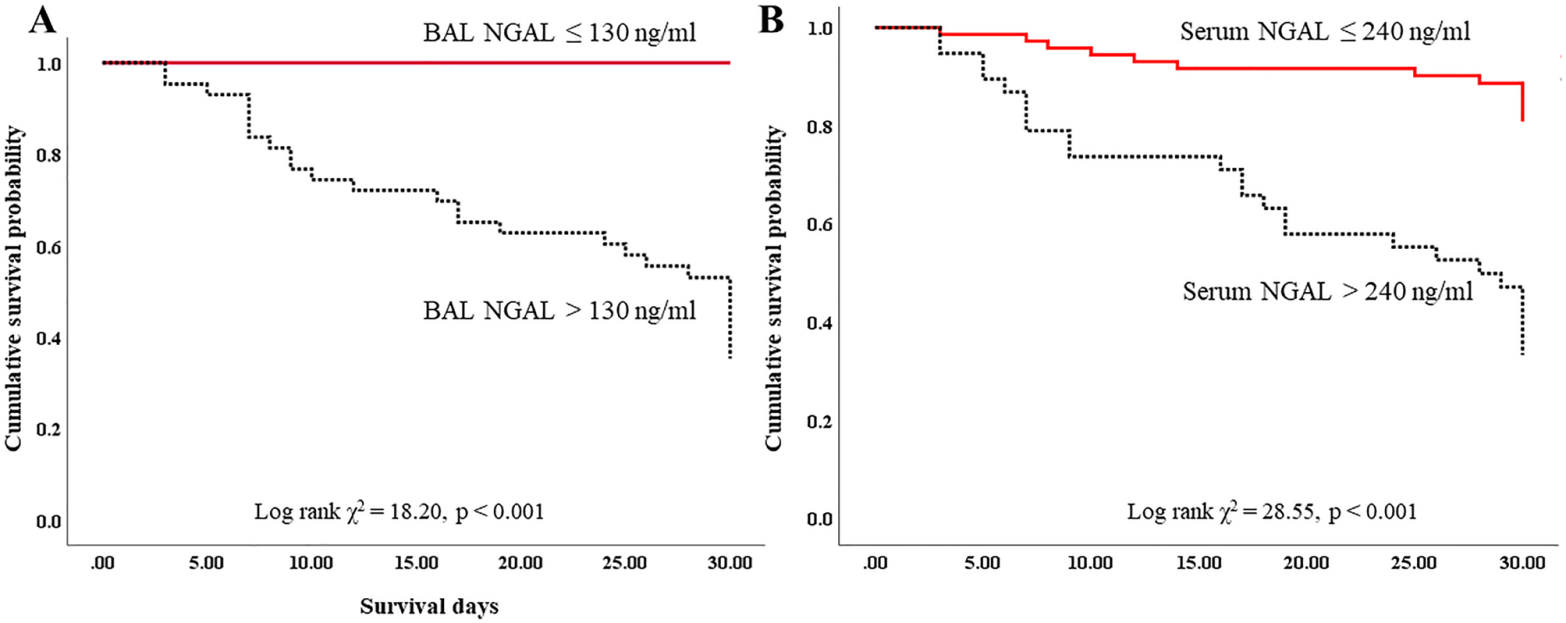
Kaplan Meier analysis for intensive care unit mortality. Patients with higher baseline BAL (**A**) or serum NGAL levels (**B**) showed a high mortality. *BAL* bronchoalveolar lavage, *NGAL* Neutrophil gelatinase-associated lipocalin.

The Cox proportional hazard model was used to evaluate the risk factors for ICU mortality. With the maximum Youden index as the cut-off point, we obtained a cut-off value of 130 ng/mL for the BAL NGAL, and 240 ng/mL for the serum NGAL. In the multivariate Cox regression analysis, RRT application during treatment (HR 3.24, 95% CI 1.47–7.10, p = 0.003), and high serum NGAL (> 240 ng/mL) (HR 5.39, 95% CI 2.67–10.85, p < 0.001) were significantly associated with ICU mortality (Table 2). However, high BAL NGAL (> 130 ng/mL) was significantly associated with ICU mortality only in univariate Cox regression analysis (Table 3). In the Kaplan–Meier analysis, a higher baseline BAL NGAL level (> 130 ng/mL) was significantly associated with higher ICU mortality (χ^2^ = 18.20, p < 0.001, Fig. 5A). In addition, a higher baseline serum NGAL level (> 240 ng/mL) was significantly associated with higher ICU mortality (χ^2^ = 28.55, p < 0.001, Fig. 5B).

**Table 2:** Cox proportional hazards regression analysis on serum NGAL for ICU mortality.

	Univariate		Multivariate	
	HR (95% CI)	P	HR (95% CI)	P
RRT during treatment	2.59 (1.26–5.32)	0.010	3.24 (1.47–7.10)	0.003
High serum NGAL (> 240 ng/ml)	4.13 (2.11–8.07)	< 0.001	5.39 (2.67–10.85)	< 0.001
Age	1.02 (1.00–1.05)	0.053		
SOFA	1.19 (1.05–1.35)	0.008		
APACHE II	1.05 (1.00–1.09)	0.041		

*HR* hazard ratio, *CI* confidence interval, *RRT* renal replacement therapy, *NGAL* neutrophil gelatinase-associated lipocalin, *SOFA* sequential organ failure assessment, *APACHE II* Acute Physiology and Chronic Health Evaluation II.

**Table 3:** Cox proportional hazards regression analysis on BAL NGAL for ICU mortality.

	Univariate		Multivariate	
	HR (95% CI)	P	HR (95% CI)	P
RRT during treatment	2.59 (1.26–5.32)	0.010	2.76 (1.04–7.36)	0.043
High BAL NGAL (> 130 ng/ml)	42.57 (1.62–1120.0)	0.025		
Age	1.02 (1.00–1.05)	0.053		
SOFA	1.19 (1.05–1.35)	0.008		
APACHE II	1.05 (1.00–1.09)	0.041		

*HR* hazard ratio, *CI* confidence interval, *RRT* renal replacement therapy, *NGAL* neutrophil gelatinase-associated lipocalin, *BAL* bronchoalveolar lavage, *SOFA* sequential organ failure assessment, *APACHE II* Acute Physiology and Chronic Health Evaluation II.

## Discussion

In this study, baseline BAL and serum NGAL levels were strongly and independently predictive of early outcomes in ARDS patients. They were significantly correlated with the initial SOFA score in patients with ARDS. ICU mortality in patients increased significantly, concurrent with the fold increase in NGAL levels compared to the controls (Supplement 1). These findings indicate that baseline NGAL levels in serum and BAL have potential as prognostic markers for ARDS. Furthermore, day 7 NGAL levels were associated with driving pressure and VFDs. The change in NGAL levels could be a novel biomarker that predicts potential alveolar epithelial injury by ARDS treatment.

Clinically, early recognition and grading of ARDS is a critical issue^17–19^. ARDS may be underestimated in clinical practice, and lack of awareness may preclude the high possibility of lung protection in the early phase of ARDS^17^. In this context, NGAL can be useful in the early identification of patients at risk, and in distinguishing patient groups for early treatment and the implementation of preventive strategies. In addition, it has the advantage of being relatively easy to measure in the emergency department, compared to scoring systems such as SOFA.

Recently, blood and urine NGAL levels have been used as an AKI biomarker, since they have an increased expression in kidney tissue after kidney ischemia^20^. However, renal impairment is a common complication in ARDS, which may confound whether serum NGAL levels reflect lung or renal tubular injury^21,22^. In this situation, BAL NGAL may more specifically reflect an origin from the lungs, as compared to serum, as BAL fluid shows a more definite estimation of lung damage, such as damage to bronchial epithelial cells or alveolar type II pnemocytes^10^. This raises the issue about the origin of BAL NGAL. Serum NGAL can leak into the alveolar space, and not necessarily through activated neutrophil, damaged alveolar, or endothelial cells. However, the correlation between BAL and serum NGAL was not significant (r = 0.224, p = 0.068), suggesting that BAL does not merely reflect the blood levels.

In this study, NGAL levels on day 7 were associated with VFDs and driving pressure. This is in line with previous studies showing a correlation between NGAL expression and the level of lung injury in VILI animal models^11,12^. Clinically, many ARDS patients remain ventilator-dependent after recovery, and VILI may contribute to mortality and morbidity in patients with ARDS^23^. Serial measurements of NGAL during the clinical course of ARDS may assist in assessing the adequacy of lung protective strategies against continued alveolar epithelial injury during ARDS treatment. Further, prospective studies are required to confirm our findings that indicate NGAL as a potential biomarker.

This study had several limitations that include its retrospective design and small sample size. This population consisted mostly of severe ARDS patients; therefore, it may be difficult to generalize our finding to all ARDS patients, including mild cases. In addition, BAL NGAL testing was not performed in some patients: those who did not undergo bronchoscopy because of hemodynamic instability at baseline; who did not consent to sample collections; or patients with insufficient samples. Although we excluded unsatisfactory samples using general clinical criteria, there is the risk of potential bias due to sampling error. Because total protein and NGAL in BAL showed the same increasing pattern in a previous study, we did not check the ratio of NGAL according to total protein or albumin^12^.

Since lung-kidney interactions play a major role in critically ill patients, there is a limitation to explaining the relevance of NGAL and ARDS without including kidney function. An increase in serum NGAL in ARDS may mean the development of organ failure such as AKI. However, we excluded patients who initially presented with AKI so as to evaluate the NGAL from a respiratory origin. Also, we checked the dynamic profile of NGAL through repeated measurements of both BAL and serum, which showed a unique correlation between lung injury and NGAL. Furthermore, NGAL predicted the prognosis of ARDS well, regardless of AKI development during treatment (Supplements 2 and 3). The novel role of NGAL in lung injury caused by mechanical ventilation using the BAL fluid of ARDS has been suggested. The discovery of this biomarker may provide a change in treatment and prediction of the prognosis in ARDS as individual approach to the ARDS. Further prospective studies are required to validate the role of NGAL as a biomarker of ARDS.

## Conclusions

The NGAL levels at baseline in serum and BAL were strongly associated with early mortality in patients with ARDS. This association persisted after adjusting for other factors, including age, severity, and RRT. Furthermore, the NGAL level at 7 days was associated with VFDs and driving pressure. Our findings suggest that the NGAL concentration may be a useful biomarker for risk stratification of ARDS, and may provide additional information on VILI in these patients. Further mechanistic studies are required to elucidate the role of NGAL in ARDS.

## Supplementary Information

Supplementary Figure 1.

Supplementary Legends.

## Data Availability

The datasets used and/or analysed during the current study are available from the corresponding author on reasonable request.

## Abbreviations

ABGA: Arterial blood gas analysis
AKI: Acute kidney injury
APACHE: Acute physiology and chronic health evaluation
ARDS: Acute respiratory distress syndrome
BAL: Bronchoalveolar lavage
BMI: Body mass index
CRP: C-reactive protein
ECMO: Extracorporeal membrane oxygenation
eGFR: Estimated glomerular filtration rate
ICU: Intensive care unit
IL: Interleukin
LIS: Lung injury score
NGAL: Neutrophil gelatinase-associated lipocalin
ROC: Receiver operating characteristic
RRT: Renal replacement therapy
SOFA: Sequential organ failure assessment
SST: Serum separate test
TNF: Tumor necrosis factor
VFD: Ventilator-free days
VILI: Ventilator-induced lung injury

## References

[1] Spadaro S (2019). Biomarkers for acute respiratory distress syndrome and prospects for personalised medicine. J. Inflamm. (Lond.)..

[2] Lb W, Ma M (2020). The acute respiratory distress syndrome. N. Engl. J. Med..

[3] Ranieri VM (2012). Acute respiratory distress syndrome: The Berlin Definition. JAMA.

[4] van der Zee P, Rietdijk W, Somhorst P, Endeman H, Gommers D (2020). A systematic review of biomarkers multivariately associated with acute respiratory distress syndrome development and mortality. Crit. Care (London, England).

[5] Huber W (2020). Prediction of outcome in patients with ARDS: A prospective cohort study comparing ARDS-definitions and other ARDS-associated parameters, ratios and scores at intubation and over time. PLoS One.

[6] Santa Cruz A (2021). Interleukin-6 is a biomarker for the development of fatal severe acute respiratory syndrome coronavirus 2 pneumonia. Front. Immunol..

[7] Yu WK (2021). Angiopoietin-2 outperforms other endothelial biomarkers associated with severe acute kidney injury in patients with severe sepsis and respiratory failure. Crit. Care (London, England).

[8] Liu C, Wang F, Cui L, Zhou J, Xu Z (2020). Diagnostic value of serum neutrophil gelatinase-associated lipocalin, interleukin-6 and anti-citrullinated alpha-enolase peptide 1 for lower respiratory tract infections. Clin. Biochem..

[9] Chen JJ, Lee TH, Lee CC, Chang CH (2021). Using lipocalin as a prognostic biomarker in acute kidney injury. Expert. Rev. Mol. Diagn..

[10] Cowland JB, Sorensen OE, Sehested M, Borregaard N (2003). Neutrophil gelatinase-associated lipocalin is up-regulated in human epithelial cells by IL-1 beta, but not by TNF-alpha. J. Immunol..

[11] Cho WH (2020). Ginsenoside ameliorated ventilator-induced lung injury in rats. J. Intensive Care.

[12] Xiao R, Chen R (2017). Neutrophil gelatinase-associated lipocalin as a potential novel biomarker for ventilator-associated lung injury. Mol. Med. Rep..

[13] Kim JW (2016). Usefulness of plasma neutrophil gelatinase-associated lipocalin concentration for predicting the severity and mortality of patients with community-acquired pneumonia. Clin. Chim. Acta.

[14] Wang J (2018). Plasma YKL-40 and NGAL are useful in distinguishing ACO from asthma and COPD. Respir. Res..

[15] Lumley A (2018). The role of neutrophil gelatinase-associated lipocalin (NGAL) in the detection of blast lung injury in a military population. J. Crit. Care.

[16] KDIGO Clinical Practice Guideline for Acute Kidney Injury (2012). Kidney Int. Suppl..

[17] Rubenfeld GD (2005). Incidence and outcomes of acute lung injury. N. Engl. J. Med..

[18] Candelaria de Haro IM-L, Eva T, Antonio A (2013). Acute respiratory distress syndrome: Prevention and early recognition. Ann. Intensive Care.

[19] Levitt JE, Matthay MA (2012). Clinical review: Early treatment of acute lung injury—Paradigm shift toward prevention and treatment prior to respiratory failure. Crit. Care.

[20] Soni SS (2010). NGAL: A biomarker of acute kidney injury and other systemic conditions. Int. Urol. Nephrol..

[21] Yeo HJ, Kim YS, Kim D, Cho ERCAWH (2020). Risk factors for complete recovery of adults after weaning from veno-venous extracorporeal membrane oxygenation for severe acute respiratory failure: An analysis from adult patients in the Extracorporeal Life Support Organization registry. J. Intensive Care.

[22] Hepokoski MEJ, Baron RM, Crotty-Alexander LE, Fuster MM, Beitler JR, Malhotra A, Singh P (2017). Ventilator-induced lung injury increases expression of endothelial inflammatory mediators in the kidney. Am. J. Physiol. Renal Physiol..

[23] Beitler JR, Malhotra A, Thompson BT (2016). Ventilator-induced lung injury. Clin. Chest Med..

