# Supplementary figures and images for: Neutrophil gelatinase-associated lipocalin as a prognostic biomarker of severe acute respiratory distress syndrome
Source: Sci Rep. 2022 May 12;12:7909. doi: 10.1038/s41598-022-12117-4 (PMC9098871; doi:10.1038/s41598-022-12117-4)

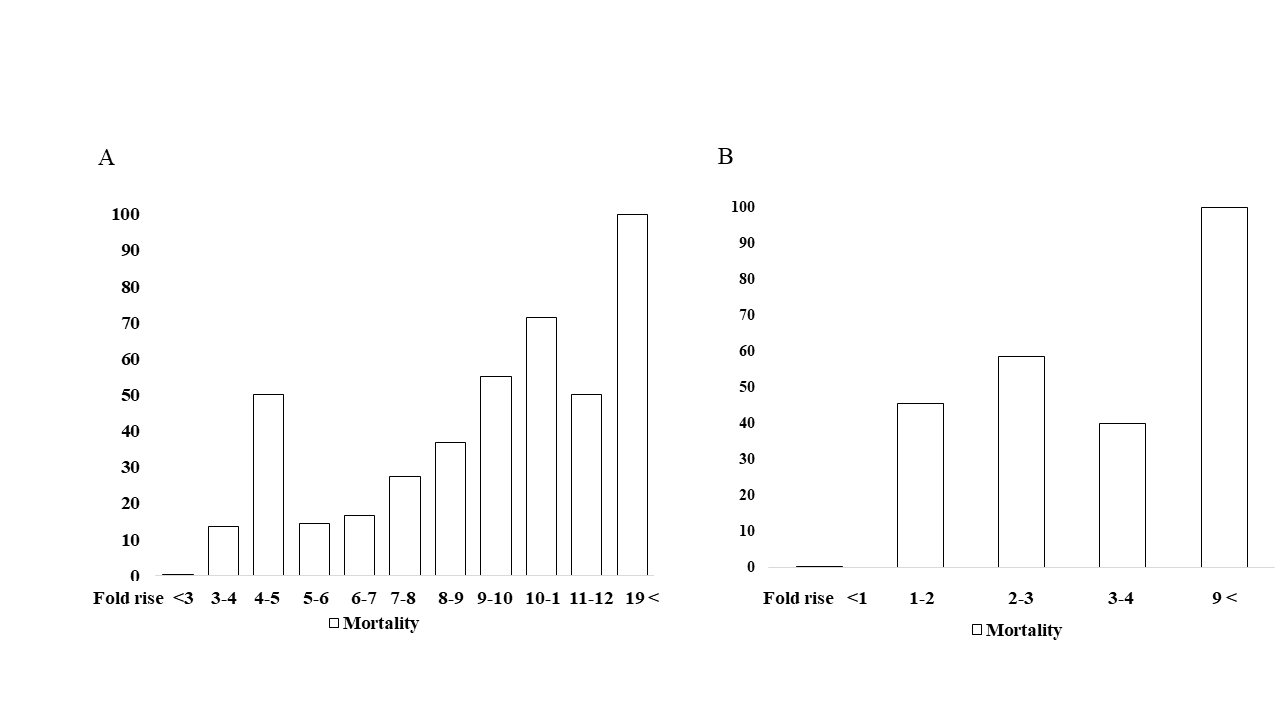

Supplement: Supplementary file 1 — Supplementary Figure 1. [file 41598_2022_12117_MOESM1_ESM.tif]
