# Supplementary material for: Neutrophil gelatinase-associated lipocalin as a prognostic biomarker of severe acute respiratory distress syndrome
Source: Sci Rep. 2022 May 12;12:7909. doi: 10.1038/s41598-022-12117-4 (PMC9098871; doi:10.1038/s41598-022-12117-4)
Supplement: Supplementary file 2 — Supplementary Legends. [file 41598_2022_12117_MOESM2_ESM.docx]

Supplementary figure 1. Distribution of intensive care unit mortality according to NGAL level

A. serum NGAL B. BAL NGAL

NGAL levels were expressed as fold increase in NGAL level (each value/mean of control)

Supplementary table 1. Cox hazard regression model for serum NGAL and ICU mortality

|  | HR (95% CI) | P |
| --- | --- | --- |
| ^*^Baseline serum NGAL | 1.09 (1.05-1.13) | <0.001 |
| Adjusting baseline eGFR | 1.09 (1.05-1.14) | <0.001 |
| Adjusting baseline eGFR, RRT during treatment | 1.10 (1.05-1.14) | <0.001 |

^*^The variables are expressed as fold increase in NGAL level (each value/mean of control).

NGAL, neutrophil gelatinase-associated lipocalin; eGFR, estimated glomerular filtration rate; RRT, renal replacement therapy

Supplementary table 2. Receiver Operating Characteristic analysis in patients except renal replacement therapy during treatment

| Variables | AUC | 95% CI | P |
| --- | --- | --- | --- |
| APACHE II | 0.491 | 0.321-0.662 | 0.920 |
| SOFA | 0.650 | 0.494-0.806 | 0.059 |
| BAL NGAL | 0.759 | 0.627-0.891 | <0.001 |
| Serum NGAL | 0.672 | 0.518-0.825 | 0.028 |

AUC, Area under the ROC Curve; CI, confidence interval; APACHE II, Acute Physiology and Chronic Health Evaluation II; SOFA, sequential organ failure assessment; BAL, bronchoalveolar lavage; NGAL, neutrophil gelatinase-associated lipocalin.
